# Geographic Distribution of CT, MRI and PET Devices in Japan: A Longitudinal Analysis Based on National Census Data

**DOI:** 10.1371/journal.pone.0126036

**Published:** 2015-05-06

**Authors:** Masatoshi Matsumoto, Soichi Koike, Saori Kashima, Kazuo Awai

**Affiliations:** 1 Department of Community-Based Medical System, Faculty of Medicine, Hiroshima University, Hiroshima, Japan; 2 Division of Health Policy and Management, Center for Community Medicine, Jichi Medical University, Tochigi, Japan; 3 Department of Public Health and Health Policy, Institute of Biomedical & Health Sciences, Hiroshima University, Hiroshima, Japan; 4 Department of Diagnostic Radiology, Institute of Biomedical & Health Sciences, Hiroshima University, Hiroshima, Japan; Leibniz Institute for Prevention Research and Epidemiology (BIPS), GERMANY

## Abstract

**Background:**

Japan has the most CT and MRI scanners per unit population in the world; however, the geographic distribution of these technologies is currently unknown. Moreover, nothing is known of the cause-effect relationship between the number of diagnostic imaging devices and their geographic distribution.

**Methods:**

Data on the number of CT, MRI and PET devices and that of their utilizations in all 1829 municipalities of Japan was generated, based on the Static Survey of Medical Institutions conducted by the government. The inter-municipality equity of the number of devices or utilizations was evaluated with Gini coefficient.

**Results:**

Between 2005 and 2011, the number of CT, MRI and PET devices in Japan increased by 47% (8789 to 12945), 19% (5034 to 5990) and 70% (274 to 466), respectively. Gini coefficient of the number of devices was largest for PET and smallest for CT (p for PET-MRI difference <0.001; MRI-CT difference <0.001). For all three modalities, Gini coefficient steadily decreased (p for 2011-2005 difference: <0.001 for CT; 0.003 for MRI; and <0.001 for PET). The number of devices in old models (single-detector CT, MRI<1.5 tesla, and conventional PET) decreased, while that in new models (multi-detector CT, MRI≥1.5 tesla, and PET-CT) increased. Gini coefficient of the old models increased or remained unchanged (increase rate of 9%, 3%, and -1%; p for 2011-2008 difference <0.001, 0.072, and 0.562, respectively), while Gini coefficient of the new models decreased (-10%, -9%, and -10%; p for 2011-2008 difference <0.001, <0.001, and <0.001 respectively). Similar results were observed in terms of utilizations.

**Conclusions:**

The more abundant a modality, the more equal the modality’s distribution. Any increase in the modality made its distribution more equal. The geographic distribution of the diagnostic imaging technology in Japan appears to be affected by spatial competition derived from a market force.

## Introduction

Japan currently has the most diagnostic imaging devices in the world. The number of computed tomography (CT) scanners per 100,000 population is 101, which is, by far, the largest among those of the Organisation for Economic Co-operation and Development (OECD) countries (Australia, with 44, is a distant second). The number of magnetic resonance imaging (MRI) scanners per 100,000 in Japan, which is 48, is also the largest among the OECD countries [1]. Despite this knowledge of device numbers, the geographic distribution of these technologies within the country is largely unknown. Few studies on their geographic distribution currently exist for any country, let alone Japan [2], despite the abundance of studies on the adoption of these technologies [3–7].

Theoretically competition shapes the distribution. In a free market, service suppliers pursue the maximization of profit. Hence, the geographic distribution of services is affected by this market force. This is the basis of the spatial competition model [8,9]. More specifically, spatial competition is an economic model in which the quantity of a service resource determines its distribution. In this model, when few service resources or suppliers exist, the distribution is skewed to cities with large populations in which the maximal profit is expected. However, an increase in service resources increases the competition for profit in the large cities. This can relocate the service resource towards smaller cities, with the result of improved equity in service distribution (the spatial competition effect) [8].

The spatial competition model is an attractive framework for considering the relationship between the amount of medical resources and its geographic distribution. However, the distribution of human resources for health, such as physicians, reportedly does not fit the prediction of this hypothesis. Even a substantial increase in the number of physicians did not improve the equity of the urban-rural distribution [10–12].

The distribution of material resources, such as diagnostic imaging devices, however, may be different from that of human resources, because, unlike humans, materials do not have the “preference for urban areas;” this preference is known to have a strong impact on human resource distribution [13–15]. The extent to which the spatial competition force influences the distribution of material resources, however, is currently unknown.

If spatial competition holds in diagnostic imaging devices, the devices would first be adopted in large cities. With an increase in numbers and subsequent competition in the large cities, they would then spread out to the smaller cities. In Japan, the first CT scanners were available in clinical practices in the 1970s; these were then followed by MRI scanners in the 1980s and positron-emission tomography (PET) scanners in the first decade of the 2000s [16,17]. Thus, PET can be regarded as being in the early state of adoption, MRI in the middle, and CT in the late stage. Thus, it is possible, in Japan, to test whether different modalities in different adoption stages illustrate different levels of equity in their distribution.

Japan is also well suited to testing the spatial competition hypothesis with regard to diagnostic imaging devices for other reasons. One of these reasons is that the entire population is covered by public health insurance, while most medical institutions, whether they be private or public, are run by profits from their practice [18]. The fee schedule for diagnostic imaging services is the same across the nation and medical institutions profit from patients’ out-of-pocket expenditures and reimbursements from public insurance. Although there are several types of insurers in Japan, the extent of services covered is uniform across the insurers. Thus there is no geographic inequity in the power to create demand for the imaging services. The second reason is because an extensive number of diagnostic imaging devices are traded every year, as the device market is large and mature in Japan. The third reason is that there is no regulation for medical institutions with regard to purchasing medical equipment [17]. As such, any hospital or clinic in any location can purchase or rent imaging devices if it can afford them. In this way, the national and local governments do not put a regional cap on the number of devices. For these reasons, it is assumed that the distribution of diagnostic imaging devices is more likely to be influenced by the market force and demand distribution in Japan, than in other countries.

Consequently, the primary purpose of this investigation is to illustrate the trend in the distribution of diagnostic imaging devices in Japan. The second purpose is to show that the geographic distribution of the devices follows a spatial competition model, by showing the association between the number of CT, MRI and PET scanners and their distributions in Japan.

## Materials and Methods

### Device data

Japan has three levels of government: municipal, prefectural and national. Data analyzed pertained to the number and use of CT, MRI and PET scanners in each of Japan’s 1829 municipalities (city, town and village). The CT, MRI and PET were chosen because they are representative of current diagnostic imaging modalities, are commonly used for scanning the entire body, and vary greatly in the timing of being available and in the amount of devices available.

Unpublicized individual data were obtained from the Static Survey of Medical Institutions that was conducted in 2005, 2008 and 2011. Permission to use the data for research was obtained by the Ministry of Health, Labour and Welfare. The Static Survey of Medical Institutions is conducted by the Ministry every three years. All clinics and hospitals in Japan are required, by national law, to report their activities and resources in the survey; the capture rate of the data in the survey was estimated, based on childbirth data, to be 91.8% in 2005, 93.8% in 2008, and 92.3% in 2011 [19]. Data on the number of CT, MRI and PET scanners in each hospital or clinic on October 1 of the year was used. Data on the number of utilizations in September of each year for CT, MRI and PET was also used. CT was classified into multi- and single-detector CT in the 2008 and 2011 survey. MRI was classified into as with <1.5 and ≥1.5 tesla; PET was classified as conventional PET and PET-CT in the 2008 and 2011 data. The information on the device versions was not available in the 2005 dataset. The 2011 survey did not cover all of the facilities in Fukushima and some of the facilities in Miyagi prefecture, because of the Great East Japan Earthquake. For this reason, data in these areas was deleted.

### Municipality data

The municipality population in 2012 was extracted from the Statistical Observations of Shi Ku, Machi, Mura 2013, which was published by the Statistics Bureau, Ministry of Internal Affairs and Communications [20]. The institution-based device data mentioned previously was connected to this municipality-based population data through the municipality code. Mergers changed the number of municipalities in Japan between 2005 and 2011, and so the 2012 municipality classification was applied to the 2005, 2008 and 2011 data.

### Statistical analysis

To obtain the transition of the number of devices, the number of devices and utilizations (performed cases) in the entire country in each year was calculated. To examine the geographic distribution of the devices, the municipalities (n = 1829) were classified into three types: “metropolis”, “city”, and “town/village”. “Metropolis” includes all of the wards (*ku*) of the ordinance-designated cities (*seirei-shitei-toshi*) and 23 special wards of Tokyo (n = 193). “City” includes the other cities (*shi*) (n = 752); “town/village” includes towns (*cho*) and villages (*son*) (n = 884). An ordinance-designated city (*seirei-shitei-toshi*) has a population greater than 500,000, which is expected to reach the 1,000,000 mark in the near future. A city (*shi*) has a population greater than 50,000. Consequently, the number of devices and their utilizations per 100,000 population in each municipality type was calculated using the data of the total number of devices or utilization and the total population in the group of municipalities.

To evaluate the inter-municipality equity of the number of devices per unit population, the Gini coefficient was calculated. The Gini coefficient is the most popular parameter of income equity. It has been used extensively in the health-related literature, in which the inter-community, or inter-facility equity of resources, have been evaluated [2,11,21–26]. In this study, we regard the imaging device as the “wealth” of the municipality and illustrate the disparity of wealth among the municipalities using the Gini coefficient. In the calculation of the Gini coefficient, all of the 1829 municipalities were ranked by the number of devices per 100,000 population. Each municipality was plotted onto the plane of coordinates with its x-axis being the cumulative proportion of the population and the y-axis being the cumulative proportion of the devices. The plotted line is the Lorenz curve; the Gini coefficient is the area between the Lorenz curve and the 45 degree line, which is divided by the triangle under the 45 degree line [11,24]. The Gini coefficient ranges from 0 (complete equity) to 1 (complete inequity), according to the variation in the number of devices per 100,000 population among the municipalities. A similar procedure was conducted for the number of utilizations. A significance test was conducted to examine the difference in the Gini coefficient between devices and between years. This was accomplished by calculating the bootstrapped standard errors for the Gini coefficient [27].

All but one of these statistical analyses were conducted using SPSS version 21 (IBM-SPSS Japan, Tokyo); the calculation of the Gini coefficients and the significance test for their differences were conducted with STATA software (version 12, College Station, TX, USA). All the maps shown in Results were created using ArcGIS version 10.0 (ESRI Japan Inc.).

### Ethics Statement

The Ethics Committee of the Graduate School of Medicine and Faculty of Medicine at the University of Tokyo has assessed and given permission for this study (assessment number 10128). The Ethics Committee for Epidemiological Research at Hiroshima University agreed to this permission (assessment number 838).

## Results

**Fig 1:**
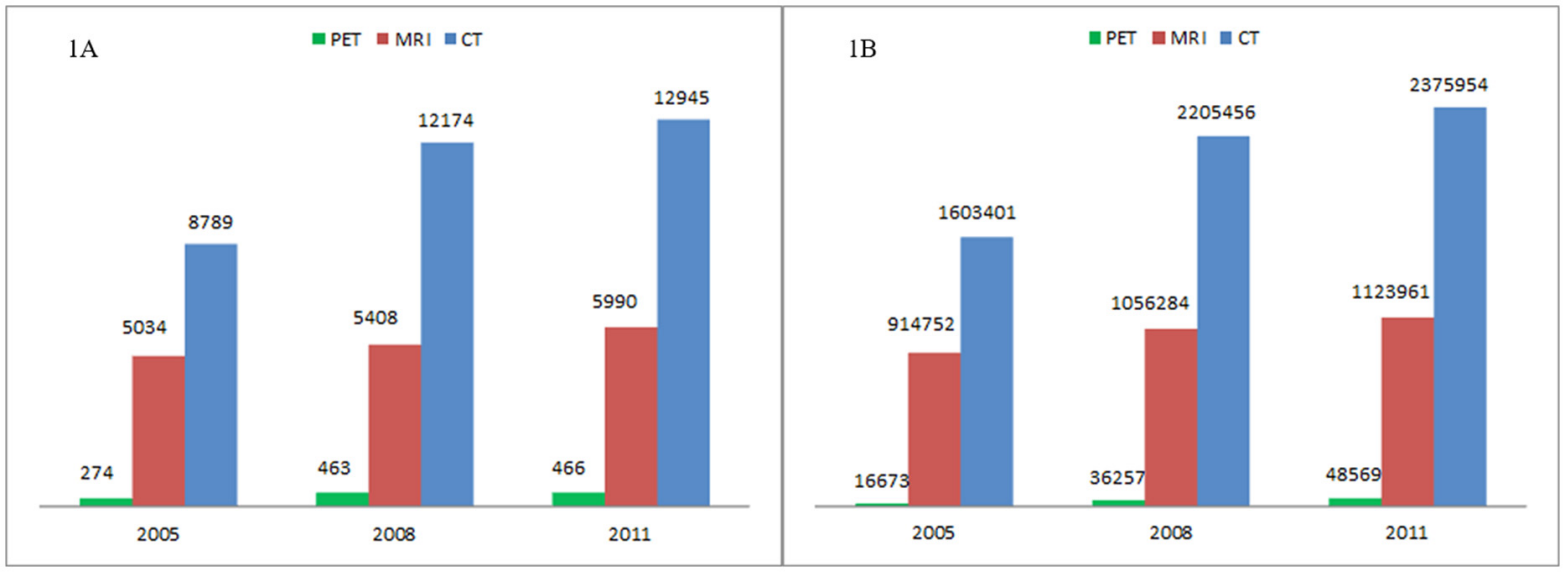
Number of devices (1A) and utilizations (1B) of CT, MRI and PET. The number of utilizations is drawn from September of each year.

The transition of the number of devices and utilizations is shown in Fig 1. Between 2005 and 2011, the number of devices and utilizations in each modality increased. The increase in the devices and utilizations is largest in the PET (70 and 191%); this was followed by the CT (47% and 48%) and MRI (19% and 23% respectively). The transition of the number of devices, and that of utilizations for each subtype of modality, is illustrated in Table 1. The number of devices, and that of utilizations for older models (i.e. single-detector CT, MRI<1.5 tesla, and conventional PET), have decreased; those for newer models (i.e. multi-detector CT, MRI ≥1.5 tesla, and PET-CT) increased. This indicates that old models were increasingly replaced by newer ones.

**Table 1:** Number of devices and utilizations classified by device version.

		Number of devices			Number of utilizations		
		2008	2011	Change (%)	2008	2011	Change (%)
CT	Single-detector CT	6231	4598	-26.2	409647	190920	-53.4
	Multi-detector CT	5943	8347	40.5	1795809	2185034	21.7
MRI	MRI <1.5 tesla	2635	2529	-4.0	316631	260950	-17.6
	MRI ≥1.5 tesla	2773	3461	24.8	739653	863011	16.7
PET	PET	199	117	-41.2	7085	6601	-6.8
	PET-CT	236	349	47.9	12850	41968	226.6

**Fig 2:**
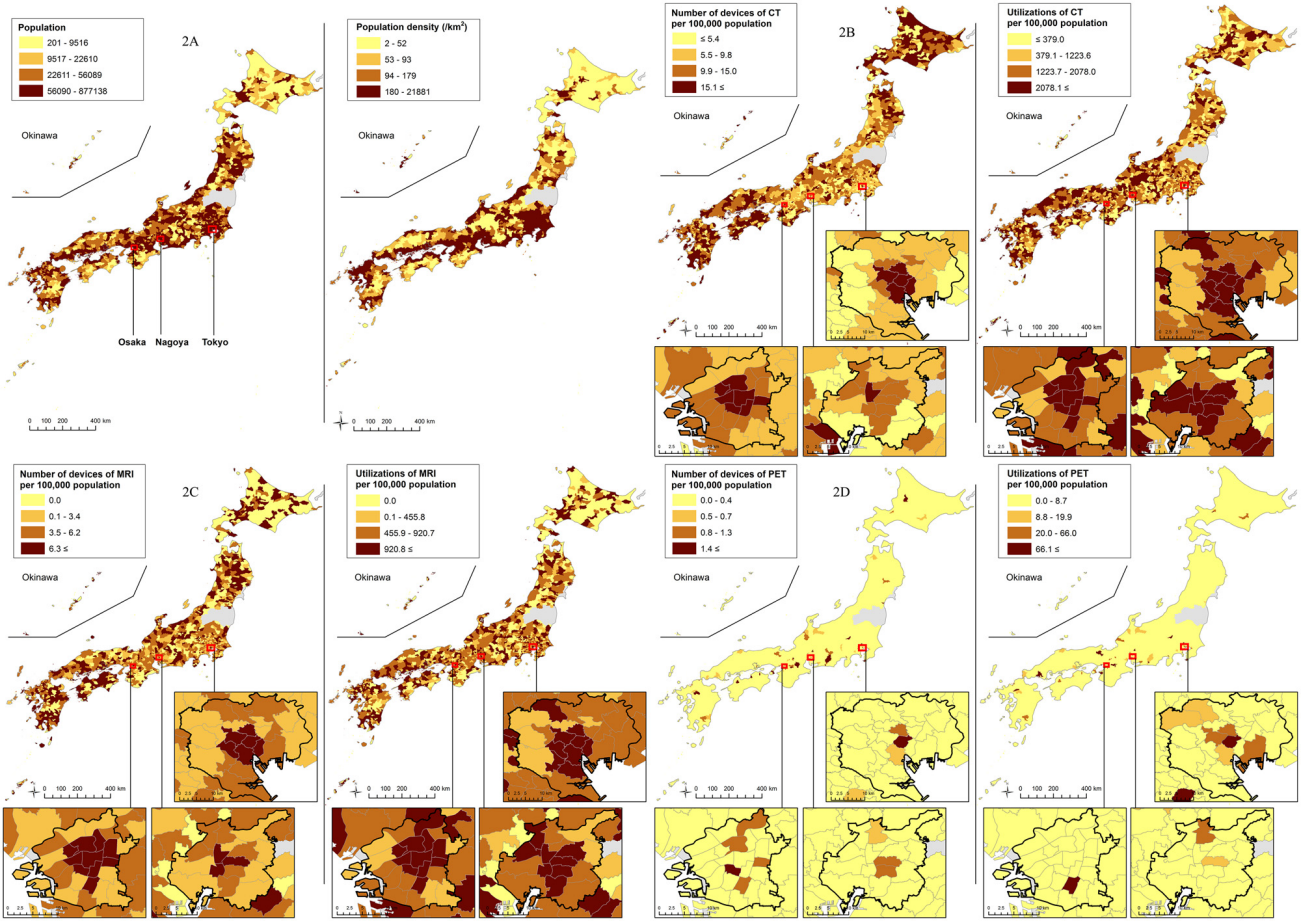
Population, population density (2A), number of devices, and number of utilizations of CT (2B), MRI (2C) and PET (2D) in each municipality in 2011. Quartile points of all values were used as cut-offs for color change except for PET in which quartile points of values excluding zero were used.

Distributions of the number of devices and utilizations per 100,000 population among the municipalities in 2011 are shown in Fig 2. Disparity was observed in the distributions of all three modalities. In particular, PET was concentrated in some limited areas, while most of the municipalities did not possess it.

**Fig 3:**
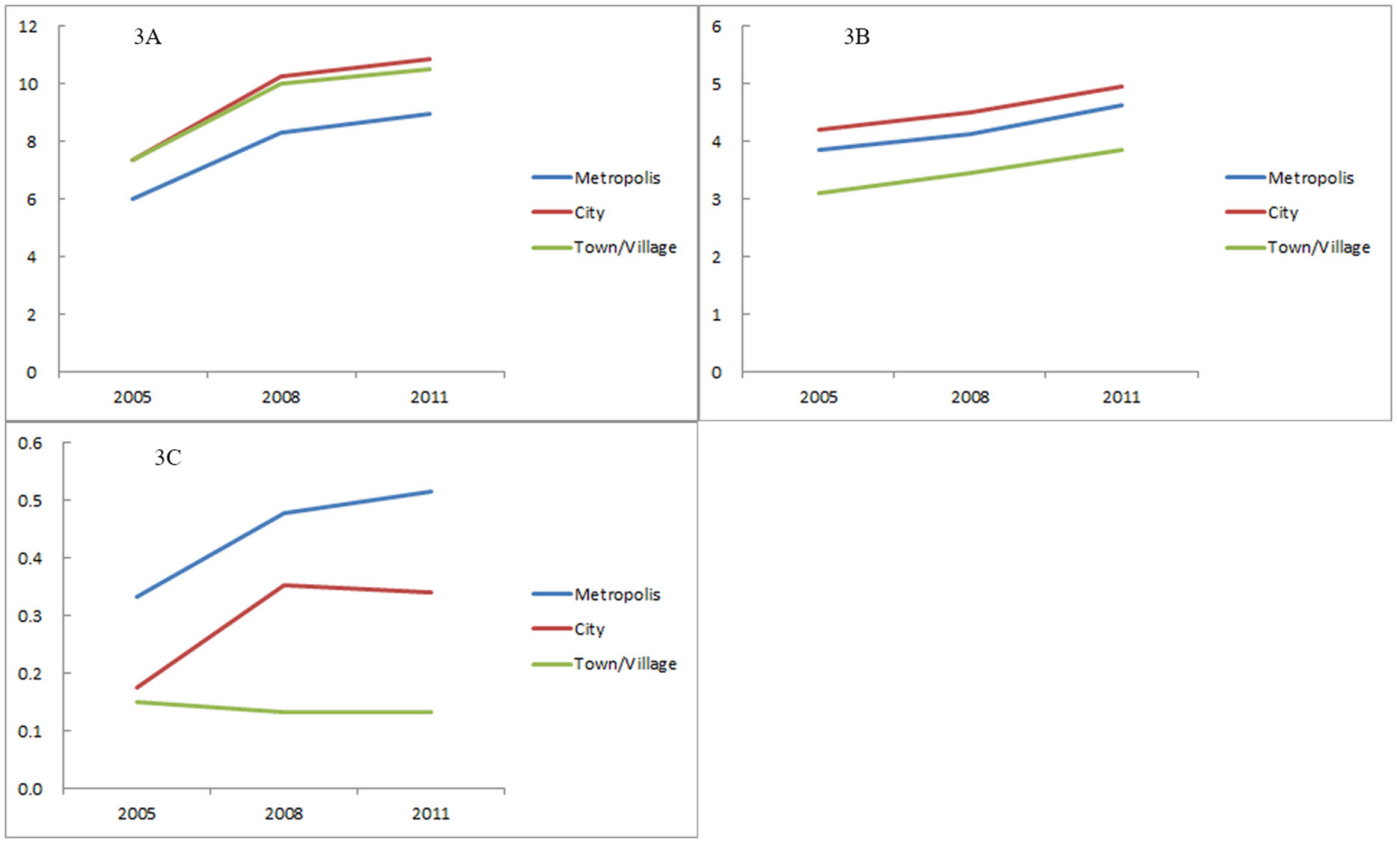
Number of devices of CT (3A), MRI (3B), and PET (3C) per 100,000 population, classified by municipality type. “Metropolis” includes all the wards (*ku*) of the ordinance-designated cities (*seirei-shitei-toshi*), as well as 23 special wards of Tokyo (n = 193). “City” includes the other cities (*shi*) (n = 752); “town/village” includes towns (*cho*) and villages (*son*) (n = 884).

**Fig 4:**
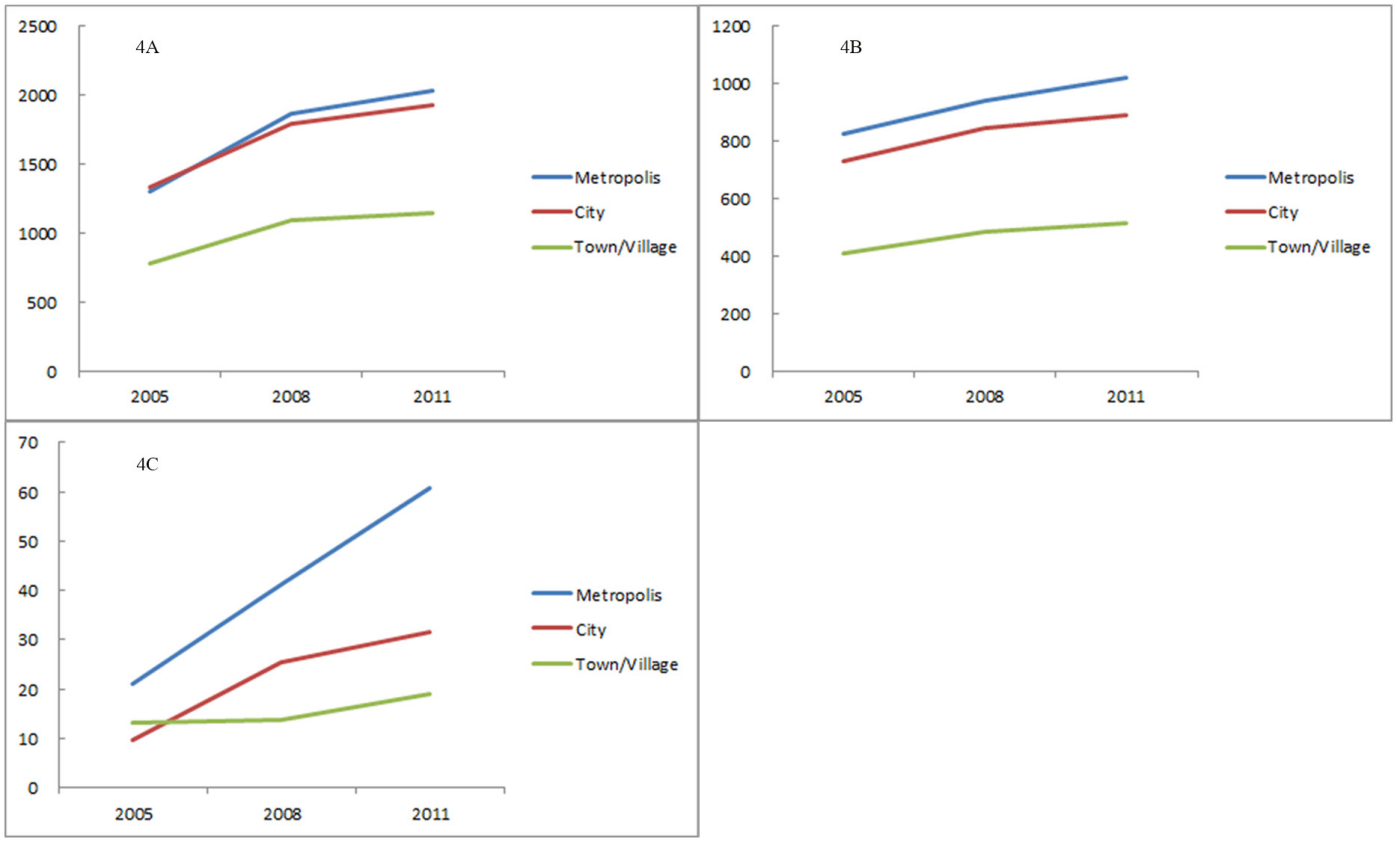
Number of utilizations of CT (4A), MRI (4B), and PET (4C) per 100,000 population, classified by municipality type.

The transition of the number of devices and of utilizations per 100,000 population by municipality type is illustrated in Figs 3 and 4. CT scanners were widespread among the municipalities, but the distribution was biased toward smaller municipalities, such as towns and villages, rather than toward metropolises. In contrast, the number of MRI scanners was higher in larger municipalities. The distribution of PET scanners was even further skewed to larger municipalities. The rate of increase of PET scanners was larger in the larger municipalities, while the increase rates of the MRI and CT scanners were not largely different among the municipality types. Compared with the distributions of devices, those of utilizations of all the three modalities were biased toward larger municipalities, which can also be observed in Fig 2 (particularly in expanded metropolises). The rate of increase in PET utilizations was larger in the larger municipalities, while the increase rates of the MRI and CT utilizations were not so different among the municipality types. For both the number of devices and utilizations, disparity among the metropolis, city and village was larger in PET than in the CT or MRI.

**Fig 5:**
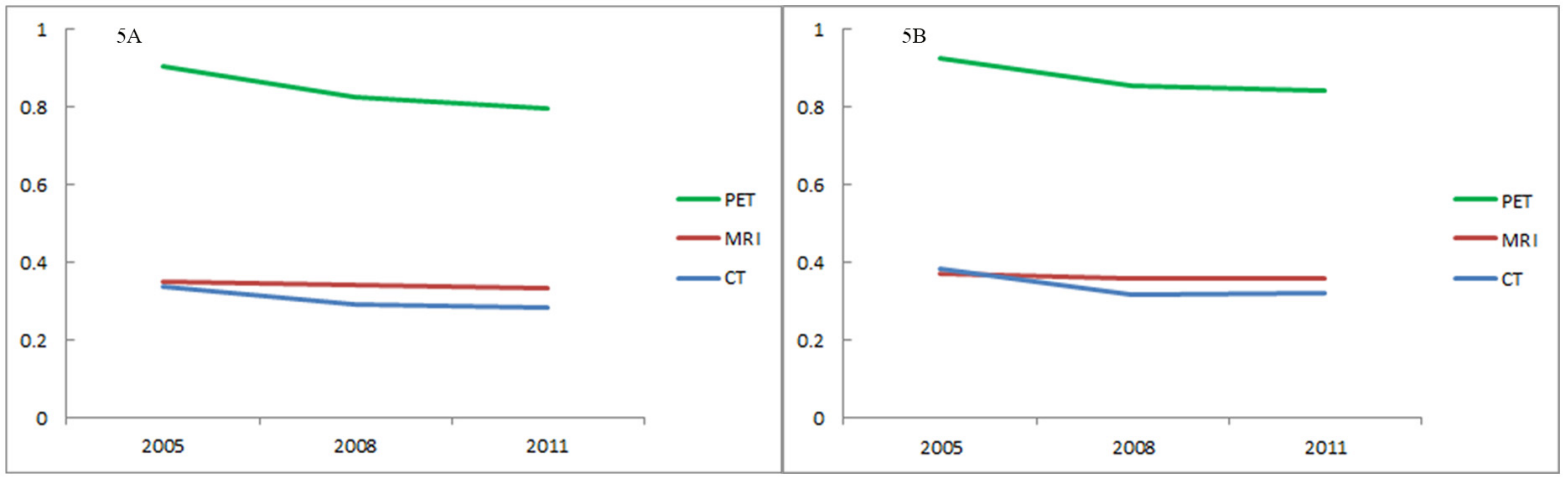
Gini coefficient of the number of devices (5A) and utilizations (5B).

The transition of the Gini coefficient for each modality is shown in Fig 5A (device) and 5B (utilization). Lorenz curves are shown as supporting information in S1 (device) and S2 Figs (utilization). The Gini coefficient of the number of devices was largest in PET and smallest in CT for any year (p for PET-MRI difference in 2008: <0.001; p for MRI-CT difference: <0.001). This indicates greater equity in the distribution of CT scanners than that of MRI or PET scanners. For all three modalities, the Gini coefficient constantly decreased for the six years (p for 2011–2005 difference: <0.001 in CT; 0.003 in MRI; and <0.001 in PET). This means an increasingly equitable distribution in all modalities. The decrease was largest in CT (16%), followed by PET (12%) and MRI (4%). The Gini coefficient of the number of utilizations illustrated a similar trend. The Gini coefficient of utilizations was largest in PET, followed by MRI and CT (p for PET-MRI difference in 2008 <0.001, p for MRI-CT difference <0.001). It did decrease (p for 2011–2005 difference <0.001 for CT; 0.003 for MRI; and <0.001 for PET) and its decrease was largest in CT (16%), followed by PET (9%) and MRI (4%).

**Fig 6:**
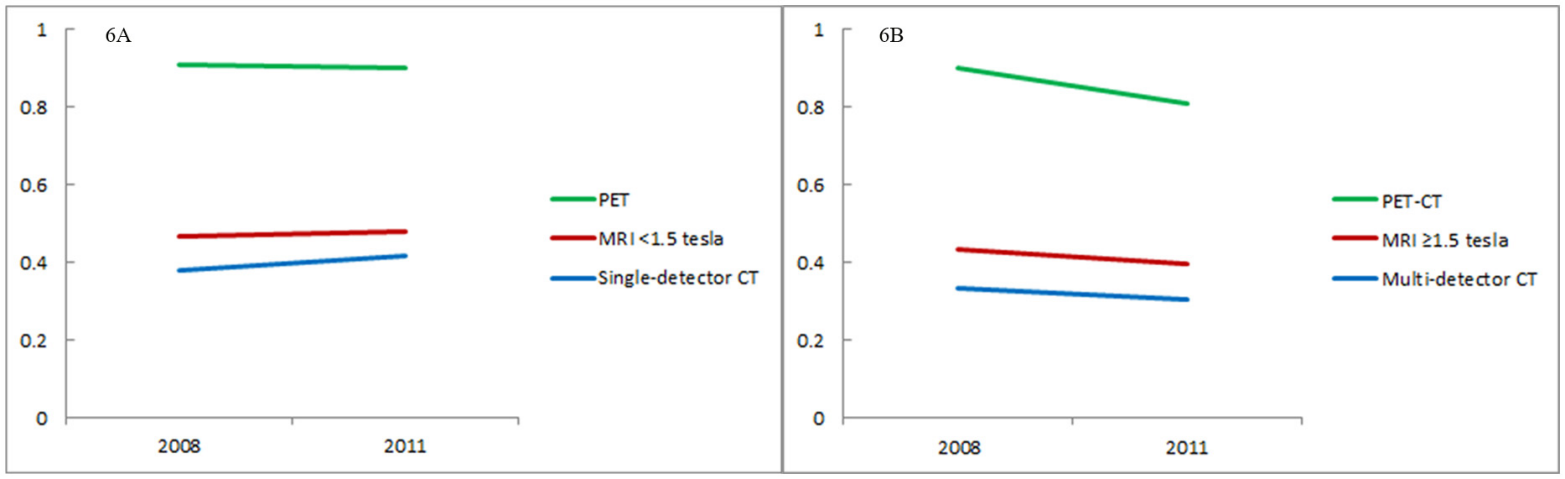
Gini coefficient of the number of older devices (6A) and newer devices (6B).

Fig 6 shows the transition of the Gini coefficient of each model with regard to the number of devices. Lorenz curves are shown as supporting information in S3 (old) and S4 (new models) Figs Both in the old and new models, the Gini coefficient was higher for the model whose number of devices was lower (p for difference between conventional PET and MRI<1.5 tesla <0.001, that between MRI<1.5 tesla and single-detector CT <0.001; that between PET-CT and MRI ≥1.5 tesla <0.001, that between MRI ≥1.5 tesla and multi-detector CT <0.001). For the three years, the Gini coefficient of old models (single-detector CT, MRI<1.5 tesla, and conventional PET) either increased or remained unchanged (increase rate 9%, 3%, and -1% respectively; p for 2011–2008 difference <0.001, 0.072, and 0.562 respectively). In contrast, the Gini coefficient of new models (multi-detector CT, MRI ≥1.5 tesla, and PET-CT) decreased (increase rate -10%, -9%, and -10% respectively; p for 2011–2008 difference <0.001, <0.001, and <0.001 respectively).

## Discussion

The results of this study illustrated that there was substantial disparity in the geographic distribution of diagnostic imaging devices in Japan. The more abundant the modality, the more equal its distribution. Increasing the number of devices or utilizations improved the equity of the distribution. Old models, which decreased in number and were less used, showed an increasingly unequal distribution, while new models, which increased in number and were used more, were distributed more equally than before.

According to the spatial competition model, a scarce resource, such as PET, highly concentrates in large cities; this creates a very unequal distribution against the population. In contrast, the distribution of an abundant resource, such as CT, becomes fairly equal against the population. An increase in the amount of resources raises the equality of its distribution, while a decrease in resources, just like that of the old models of the devices, reduces the equality. All of these predictions were observed in the results. Thus, the results suggest that the relationship between the amount and the distribution of technology resources is influenced by spatial competition.

Among all of the types of service resources, the geographic distribution of physicians has been most extensively studied. In Japan, the number of physicians per unit population has increased by 70% over the past 30 years. That being said, the equity of their geographic distribution has remained unchanged [10–12,28–30]. A similar trend was observed in the United States [10,31]. The geographic distribution of physicians thus does not appear to follow the spatial competition model. Some of the proposed reasons for this are the physician’s preference for urban areas and urban-rural imbalance of the physician’s background [14,32,33]. Material resources do not have such elements. Thus, the distribution of diagnostic imaging devices is supposed to be influenced more directly by market forces than that of human resources. Hospitals and clinics in Japan, whether located in urban or rural areas, can possess diagnostic imaging devices if there is sufficient demand and if the institutions can afford to rent or buy them. This factor would separate the distribution pattern between human and material resources.

As illustrated in Figs 3 and 4, the distribution of CT devices was skewed to rural municipalities, while that of CT utilizations was biased towards urban municipalities. This gap may be caused by the unique health care environment in Japan. Japanese health care is a combination of public and private institutions. The proportion of public institutions is higher in the rural areas, than in the urban areas [34]. Public medical institutions are run by profit from their practice, as well as with a local or national governmental budget. In addition, public and private medical institutions in rural areas can purchase equipment with the help of state subsidies. Thus, a rural medical institution is probably more likely to possess expensive diagnostic imaging devices than an urban institution if the expected profits gained from using the devices are the same [35]. This can increase the number of imaging devices in rural areas, particularly devices usable at primary care facilities, such as CT, while their utilizations are relatively small.

### Limitations

The financial protection policy for rural medical institutions potentially influenced the snapshot distribution of the devices in each year. This can lead to an overestimation of the spatial competition effect at each time point by biasing the distribution toward rural areas. In this sense, a comparison of the Gini coefficients among CT, MRI and PET at a certain time needs attention. However, the longitudinal change of the equity of the distribution, in response to the change in the amount of the devices, which is the primary reason for the existence of spatial competition, is not affected by this policy.

The rural protection policy could also be reflected in the distribution pattern of the subtypes of the modalities. As shown in the results, the number of new models of CT, MRI and PET increased and spread increasingly to small municipalities, while the number of old models decreased and their distributions gravitated towards large municipalities. This suggests that the replacement of old models with new ones, or the installation of new models in addition to conventional ones, are more common in rural than in urban areas in Japan.

MRI, and PET, in particular, cannot be placed in rural facilities without full-time radiologists, because the use of this equipment requires highly specialized knowledge. In Japan, most institutes with PET scanners only perform ^18^F-Fluorodeoxyglucose (^18^F-FDG)-PET examinations and use ^18^F-FDG compounds delivered from commercially available PET laboratories. Because the half-life period of ^18^F-FDG is about 110 minutes, institutions using delivery ^18^F-FDG compounds must be located close to the PET laboratories. When comparing the equity of the distribution among CT, MRI and PET, both the spatial competition effect and these factors should be taken into account.

The number of utilizations in this study is for only one month, which is less reliable than the annual number. In addition, the CT data in the Static Survey of Medical Institutions in 2005 is not that of all types of CT, but only that of helical CT. Thus, it needs attention when 2005 CT data is compared with CT data in other years. However, by 2005, most of the conventional CT scanners were presumably replaced by the helical model in Japan. Thus, we consider that the difference between our data and the real number of CT scanners was small.

The nature of the Gini coefficients is also an issue. The Gini coefficient is most sensitive to the shape of the middle part of the Lorenz curve [21,36]. In our data, most municipalities did not possess PET. As such, the Lorenz curve of PET is a flat line in the low to middle part of the spectrum, rather than a true curve. In this situation, inequity illustrated by the Gini coefficient may be overestimated, as compared with that of CT and MRI. In addition, the Gini coefficient can only be interpreted relatively, because there is no clear definition of a low or high value.

## Conclusions

A reasonable allocation of medical resources, based on the distribution of demand, is one focus of political interventions. The uneven distribution of human resources, such as physicians, is a worldwide problem. Consequently, numerous policies, sometimes using state power and a substantial budget, have been implemented to address this issue [33,37,38]. Although advanced medical equipment, such as MRI and PET, should be allowed to be concentrated in large cities because of its scarcity, devices which are regarded in Japan as tools for primary care, such as X-ray machines and CT, need to be distributed equally. The distribution of diagnostic imaging devices seems to be directly influenced by the invisible hand of the market, and thus is more likely to be optimized by an increase in numbers as compared with the distribution of human resource. Hence, we recommend, with regard to devices that need to be distributed fairly, that political priority be placed on securing resources in the right quantities, instead of in the allocation of resources.

## Supporting Information

S1 Fig Lorenz curves of the number of CT, MRI, and PET devices. (TIF)

S2 Fig Lorenz curves of the number of CT, MRI, and PET utilizations. (TIF)

S3 Fig Lorenz curves of the number of single-detector CT, MRI<1.5 tesla, and conventional PET devices. (TIF)

S4 Fig Lorenz curves of the number of multi-detector CT, MRI ≥1.5 tesla, and PET-CT devices. (TIF)
