# Supplementary figures and images for: Geographic Distribution of CT, MRI and PET Devices in Japan: A Longitudinal Analysis Based on National Census Data
Source: PLoS One. 2015 May 6;10(5):e0126036. doi: 10.1371/journal.pone.0126036 (PMC4422695; doi:10.1371/journal.pone.0126036)

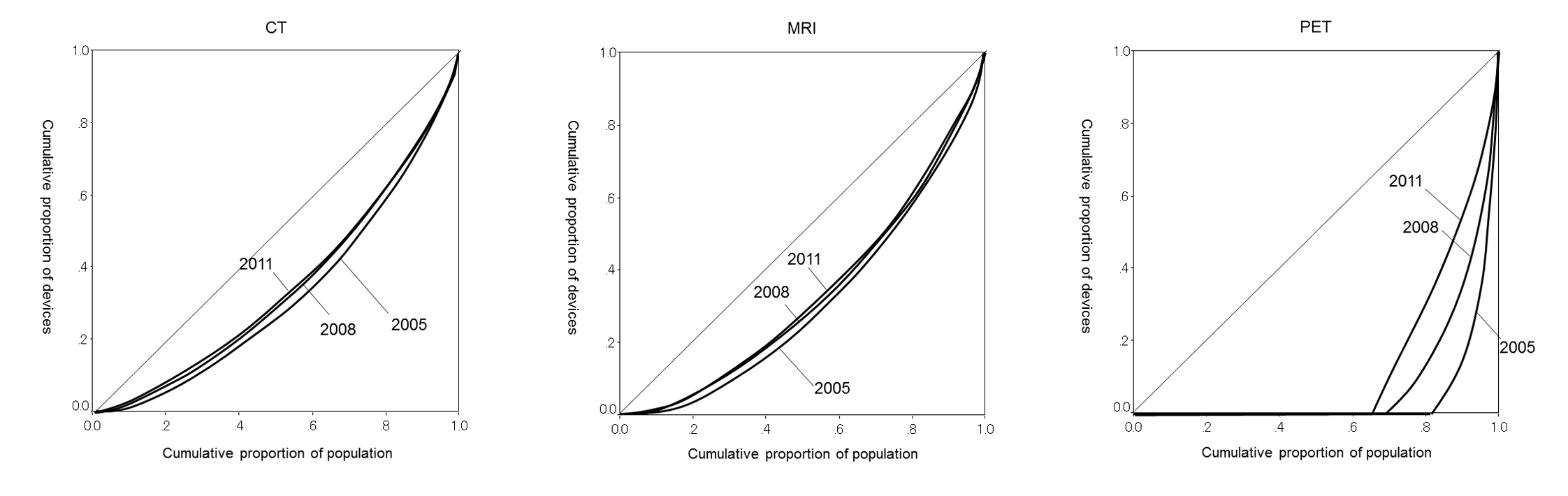

Supplement: S1 Fig — (TIF) [file pone.0126036.s001.tif]

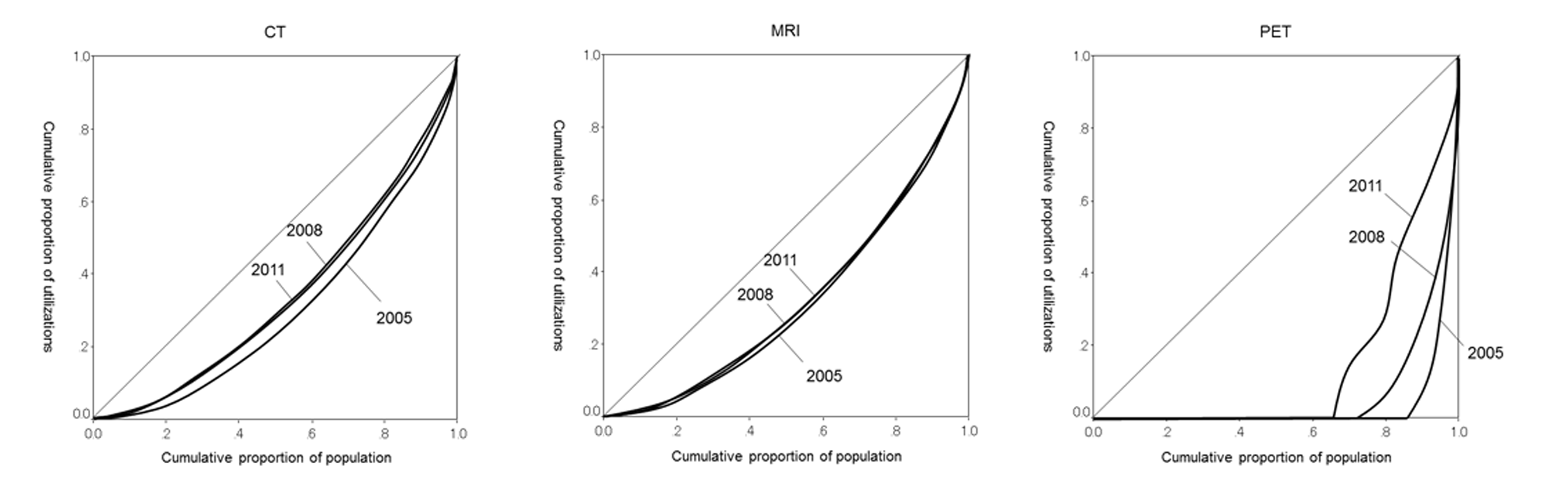

Supplement: S2 Fig — (TIF) [file pone.0126036.s002.tif]

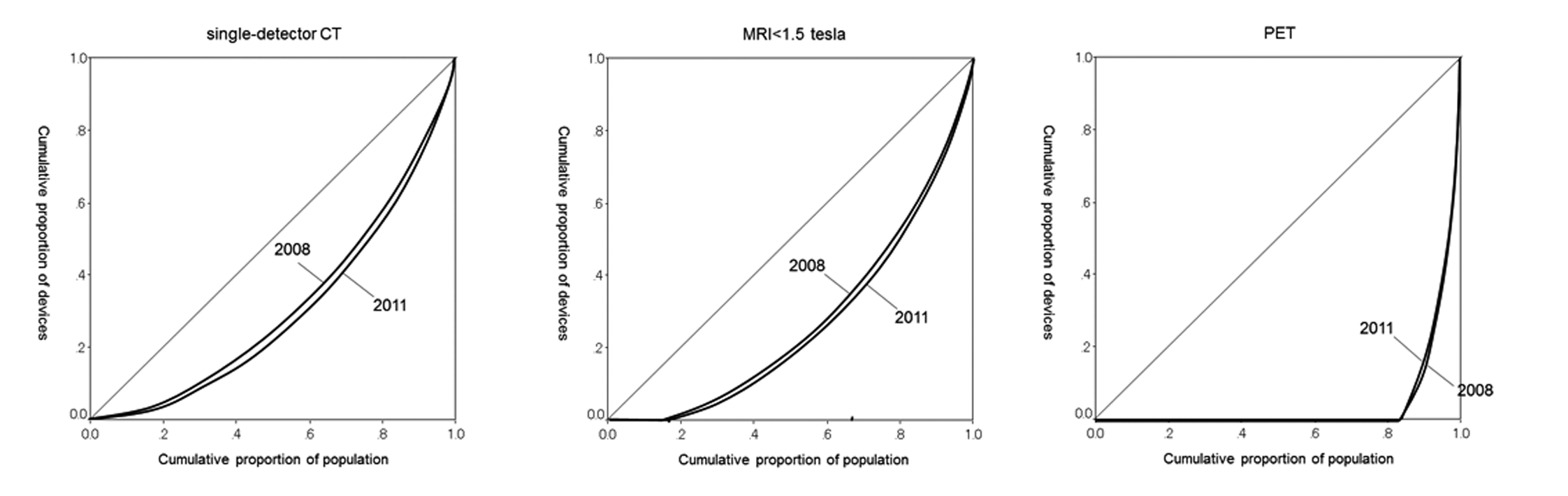

Supplement: S3 Fig — (TIF) [file pone.0126036.s003.tif]

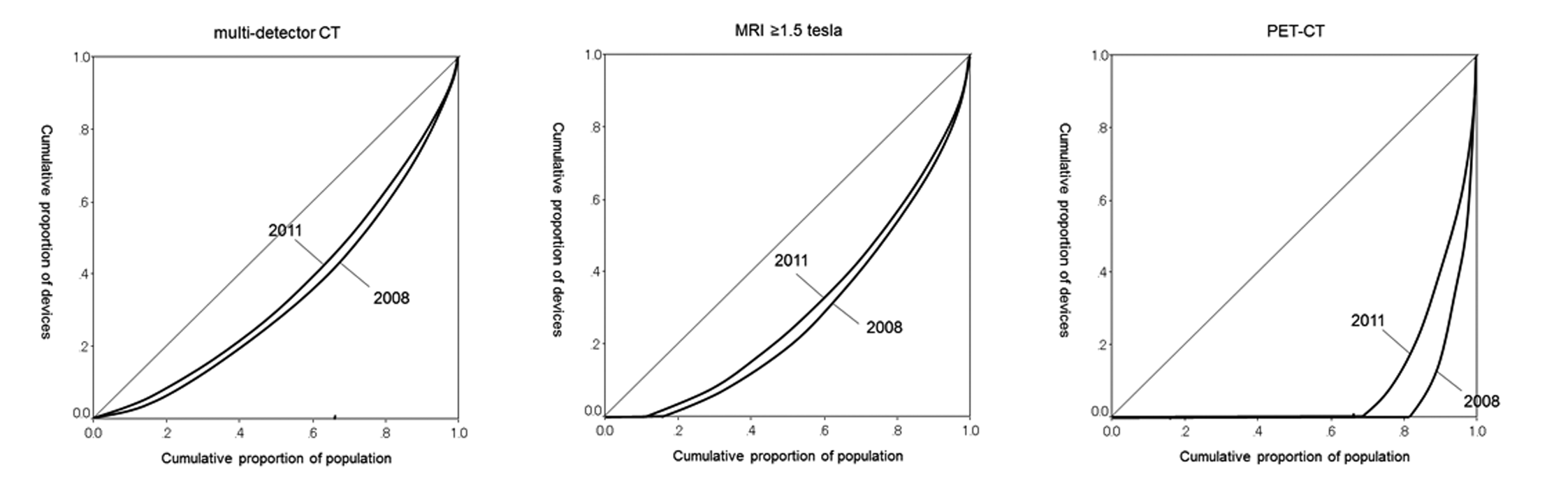

Supplement: S4 Fig — (TIF) [file pone.0126036.s004.tif]
